# *Natrix natrix* after dark: citizen science sheds light on the common grass snake’s nightlife

**DOI:** 10.7717/peerj.17168

**Published:** 2024-04-25

**Authors:** Petronel Spaseni, Tiberiu C. Sahlean, Iulian Gherghel, Ștefan R. Zamfirescu, Ionuț C. Petreanu, Raluca Melenciuc, Cristina F. Alistar, Viorel D. Gavril, Alexandru Strugariu

**Affiliations:** 1 Department of Exact and Natural Sciences, Institute of Interdisciplinary Research, Alexandru Ioan Cuza University of Iași, Iași, Iași, Romania; 2 Faculty of Biology, Alexandru Ioan Cuza University of Iași, Iași, Iași, Romania; 3 Institute of Biology Bucharest, Romanian Academy, Bucharest, Bucharest, Romania; 4 Faculty of Natural and Agricultural Sciences, Ovidius University of Constanţa, Constanța, Constanța, Romania; 5 Department of Biochemistry and Molecular Biology, Faculty of Biology, University of Bucharest, Bucharest, Bucharest, Romania

**Keywords:** Ectotherms, Reptiles, Activity patterns, Nocturnal activity, Crepuscular activity, iNaturalist, Europe, Color polymorphism

## Abstract

Activity patterns in animals are often species-specific, and can be generally categorized as diurnal, crepuscular, or nocturnal. Understanding these patterns provides insight into ecological adaptations and behaviors. The common grass snake (*Natrix natrix*), one of the most common and widespread European snake species, is traditionally considered diurnal, with scarce evidence of its crepuscular and nocturnal activity. We aimed to document the distribution, environmental conditions, and potential phenotype associations of nighttime activity in *N. natrix*. We used citizen science data from iNaturalist (1992–2022), Observation.org (2012–2022), together with personal field observations (2010–2023) to collect 127 crepuscular and nocturnal activity records. Most observations occurred between May and August, coinciding with the peak activity period of grass snakes across their distribution range. Statistical analyses revealed no significant difference in mean daily temperatures between crepuscular and nocturnal observations. However, striped individuals displayed nocturnal activity at higher temperatures, consistent with their distribution in warmer regions, but failed to register any difference when tested on a geographic subsample, that accounted for sympatry of the phenotypes. Surprisingly, we found no significant impact of moon presence or moonlight on nighttime activity or age class, contrary to expectations based on other snake species’ responses. While our study reveals that nocturnal activity in the common grass snake is geographically widespread, further research is warranted to understand its drivers and ecological implications. This study highlights the value of citizen science platforms for biological and ecological research, offering unparalleled spatial and temporal coverage by their users. In conclusion, our work extends the knowledge of nocturnal behavior in *N. natrix* and underlines the critical role of citizen science in discovering behavioral aspects of common and widespread species.

## Introduction

Activity patterns in animals are typically deeply ingrained within species, as most animals adhere to specific activity times (Abom et al., 2012). In broad terms, species can generally be categorized as diurnal (active during the day), crepuscular (active at dawn and dusk), or nocturnal (active during the night) (Metcalfe, Fraser & Burns, 1998). Nocturnal activity is an important aspect of a species’ biology, and it is present in every major animal group (Saigusa & Oishi, 2000; Gerkema et al., 2013; Ward et al., 2014; Meiri, 2018; Levy et al., 2019). Nocturnal animals are primarily active during the nighttime, when most of their necessary activities are done (*e.g.*, foraging for resources, feeding, mating *etc*.). The factors that influence nocturnality may vary among species, but the main ones are competition for resources (Kronfeld-Schor & Dayan, 2003), avoidance or enhancement of predation (Ross et al., 2013), and thermal constraints (Brivio et al., 2024). Amphibians can be active at lower body temperatures, making nocturnality quite common, with humidity and precipitation being the defining factors for such behaviors (Vitt & Caldwell, 2014). By contrast, fluctuations in environmental temperatures strongly influence the diel and seasonal activity of reptiles (Vitt & Caldwell, 2014). This defines their metabolic and foraging rate or the ability to avoid predation (Papastamatiou et al., 2015).

Snakes display a variety of daily activity patterns that can be further categorized in two major groups, those related to thermoregulation and temperature relationships, and those associated with the time of day when the activity occurs (diel patterns) (Seigel, Collins & Novak, 1987). Many species from very arid habitats are nocturnal (Sivan et al., 2013; Signore, Clark & Schraft, 2022) to avoid extreme heat during the day (Whitaker & Shine, 2002) and to reduce water loss (Ladyman & Bradshaw, 2003). Nocturnal behavior is also common among most tropical species, where it is associated with higher relative humidity during the night, that can help with water conservation and feeding success (Daltry et al., 1998; Brown, Shine & Madsen, 2005). Additionally, there are species with temporal flexibility; these snakes can shift their daily activity patterns seasonally in response to fluctuating environmental temperatures (Abom et al., 2012; DeGregorio et al., 2014). When looking at the activity patterns of European snakes, most species are diurnal, with only a small number of taxa being nocturnal (*e.g.*, *Eryx jaculus, Telescopus fallax*), and about the same percentage exhibiting a mix of diurnal and nocturnal diel patterns (Speybroeck et al., 2016).

In American natricine snakes, nocturnal activity is associated with season, ambiental, and body temperature (Mushinsky, Hebrard & Walley, 1980; Sievert & Andreadis, 1999; Brown & Weatherhead, 2000). Individuals active during the night have also been reported in European *Natrix* species, especially from around the Mediterranean region. Viperine water snakes (*Natrix maura*) have been observed in greater numbers at night during June and July in Italy (Scali, 2011) and across the whole activity season in Spain (Hailey & Davies, 1987; Jaén-Peña & Pérez-Mellado, 1989). In dice snakes (*Natrix tessellata*), nocturnal behavior is more widespread, with records from Italy (Scali, Dimitolo & Montonati, 2001; Scali, 2011), Greece (Mebert et al., 2011), Russia (Tuniyev et al., 2011), Bulgaria (Moller, 1990), Hungary (Kreiner, 2007), and Switzerland (Mebert et al., 2011).

The grass snake, *Natrix natrix* sensu lato, is a sister taxon to the viperine water snake and the dice snake. Previously considered a polytypic species, current evidence suggests the existence of three different species: the red-eyed grass snake *N. astreptophora* (Seoane, 1884), the barred grass snake *N. helvetica* (Lacepède, 1789), and the common grass snake *N. natrix* (Linnaeus, 1758) (Speybroeck et al., 2020). Our study concerns the nominate species (*Natrix natrix* sensu stricto according to current classifications) which, depending on the subspecies, can reach a total length of 150 to 200 cm and feeds on amphibians, fish and small mammals or birds (Fuhn & Vancea, 1961; Kindler et al., 2013; Speybroeck et al., 2016; Asztalos et al., 2021). Throughout its range, from the Rhine region in Germany eastward to Lake Baikal, including also Fennoscandia, the Balkan Peninsula and some parts of the Middle East (Kindler et al., 2017; Fritz & Schmidtler, 2020; Asztalos et al., 2021), the grass snake is mostly associated with aquatic habitats.

The common grass snake is also a color-polymorphic species, with several discrete phenotypes being known. The common morph is characterized by olive-green, brown or greyish dorsal color, frequently with an inconspicuous body pattern (absent or small dark spots), and it is the most widespread, being the dominant form in most parts of the species range; the striped morph is predominantly found in the Balkan Peninsula, where it is usually the dominant morph, while melanistic individuals have been, mostly sporadically, reported from throughout the species’ range. While most populations are monomorphic (with either the common morph, or the striped morph, being present), a few populations comprise two or even three morphs (common, striped, and melanistic) (Fănaru et al., 2022). Color polymorphism in reptiles has bennefited from intense ecological and evolutionary research over the past decades (Forsman & Ås, 1987; Clusella Trullas, Van Wyk & Spotila, 2007; Geen & Johnston, 2014; Martínez-Freiría et al., 2017; Santos et al., 2018), as coloration can play important roles in natural and sexual selection, and may be particularly relevant for thermoregulation, habitat use and activity patterns in ectotherms (Gibson & Falls, 1979; Muri et al., 2015; Martínez-Freiría et al., 2020). Nevertheless, this aspect has barely been investigated in grass snakes, with some studies exploring the effects of melanism on body size and sex ratio (Bury et al., 2020; Fănaru et al., 2022), while others have focused on the distribution of coloration and patterns (Fritz & Ihlow, 2022).

In the absence of exhaustive systematically collected biological data, citizen science has been proven to be a highly useful tool for exploring a plethora of subjects, using the public sector to facilitate large-scale data collection. This can enhance our knowledge of biodiversity and ecosystems, particularly the spatial distribution of various organisms (Sullivan et al., 2009; Unger et al., 2021; Pintilioaie, Sfîcă & Baltag, 2023). Moreover, it has been important in providing insights into different behavioral patterns and unusual life history traits of various species (Kobori et al., 2016; Blais & Shaw, 2018), as well as a basis for more rigorous testing of ecological hypotheses (Moore et al., 2019; Fritz & Ihlow, 2022).

Although *N. natrix* is considered both diurnal and nocturnal (Speybroeck et al., 2016), there is very limited empirical data available to support this. While some publications mention nocturnality of *N. natrix sensu lato*, one of them concerns the Sardinian grass snake (Capula, Rugiero & Luiselli, 1994), which is now regarded as a different species (Schultze et al., 2020; Fritz & Schmidtler, 2020), while others provide little to no evidence for this phenomenon (Agrimi & Luiselli, 1994; Mertens, 1994; Speybroeck et al., 2016). Here we aimed to document the patterns of crepuscular and nocturnal activity in *N. natrix* across its distribution range using citizen science, as well as our own data, in order to understand (i) how common it is, (ii) whether there are any geographical variations, (iii) whether there is any significant association between environmental variables (temperature, and moon presence and moonlight) and the activity patterns, and (iv) whether there is any significant difference regarding temperatures between the phenotype (color morph) in crepuscular and nocturnal snakes. Thus, we expect moon presence and illumination to be associated with age class (as adults may take higher risks, as to an increase in prey availability). We also expect that nocturnal observations will happen at higher mean temperatures than crepuscular ones, since we predict that higher temperatures during the day will lead to more suitable thermal conditions later into the night. Finally, we expect crepuscular and nocturnal striped grass snakes to be active at higher average temperatures than the other morphs due to their more southern distribution, where the climate is milder.

## Materials & Methods

### Occurrence records

We obtained 9,745 observational data of *N. natrix* from the iNaturalist database (Data S1). We used the *rinat* package (Barve & Hart, 2023) available for R v4.2.2 (R Core Team, 2022) to download all georeferenced records present by February 22nd, 2023. In order to distinguish observations of nighttime activity, we followed the protocol developed by Dyugmedzhiev et al. (2020) and identified crepuscular activity as occurring 15 min before sunset to 30 min after, and nocturnal activity as occurring more than 30 min after sunset. We used the *suncalc* package (Thieurmel & Elmarhraoui, 2022) to compute sunrise, sunset, moonrise, and moonset times for every observation. To select only crepuscular and nocturnal observations we used the before-mentioned constraints to compare the time of observations and the obtained sunset times. We also manually verified all the records. This was necessary because of timestamp or data entry errors. Night observations of dead individuals were omitted since the time of death could not be determined. To do this we used a blind-validating system with three observers, where each observer was asked to confirm whether an observation corresponded to crepuscular or nocturnal activity. Additionally, we included authors’ personal opportunistic observations of nighttime activity for the grass snakes, which were recorded during various field surveys carried throughout Europe (mostly Romania) between 2010–2023. Finally, we manually verified records from Observation.org that included a time of observation and photographs. We used the filters available on the webpage for this task, and we extracted only the records that were realized in the late afternoon or during the night based on the time of observation We then applied the same procedure as for iNaturalist data, to check if they belonged to one of the categories of nighttime activity or not, prior to adding them to the final database.

**Figure 1:**
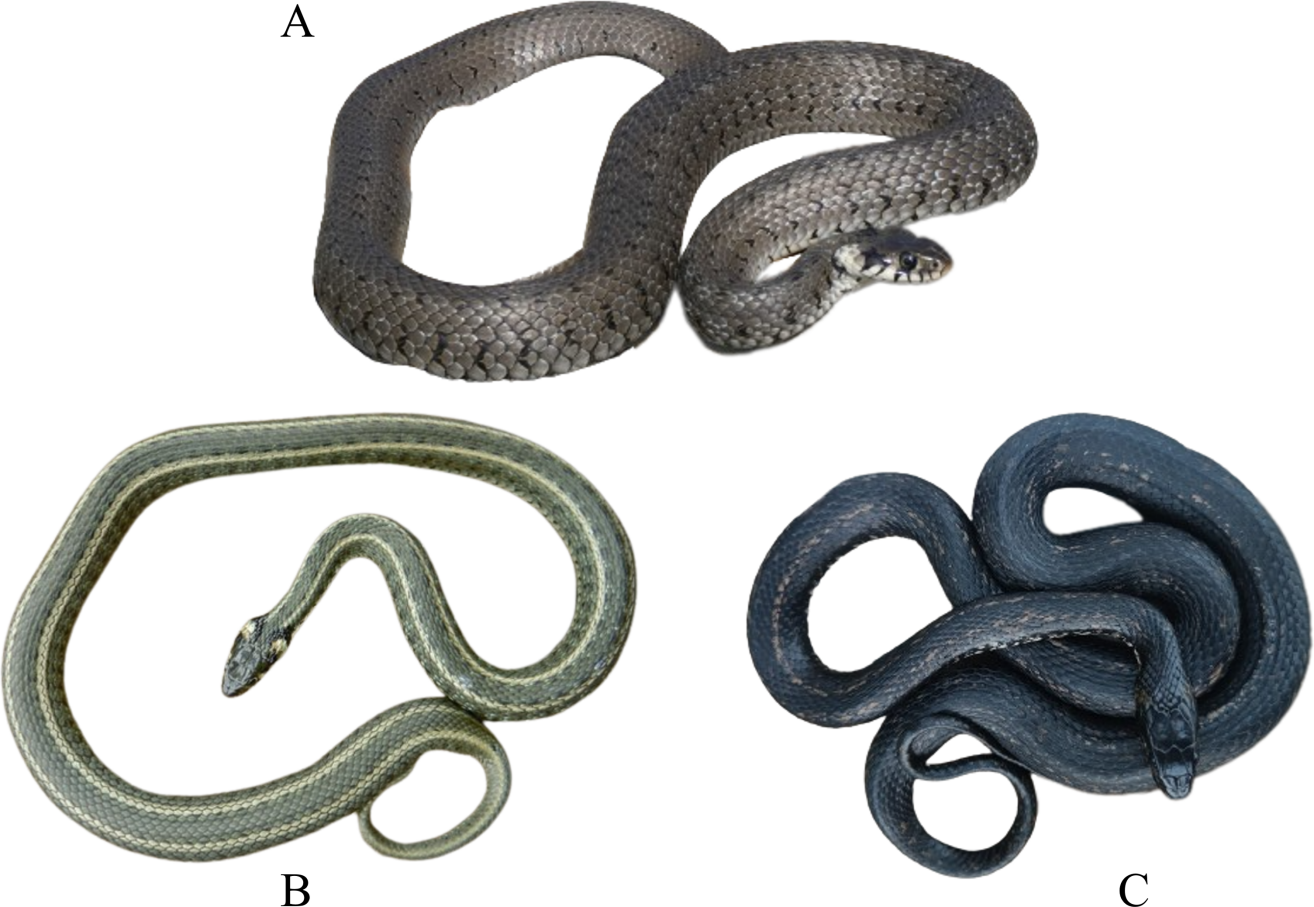
Examples of body pattern phenotypes of *Natrix natrix*. (A) Dorsal view of an unstriped individual (photo credit: I Gherghel); (B) dorsal view of a striped individual (photo credit: A Strugariu); C. dorsal view of a melanistic individual (photo credit: A Jurjescu).

For every data point we considered information regarding body pattern (striped, common, and melanistic) (Fig. 1), age class (immature or adult), number of individuals, and class of habitat (aquatic or terrestrial). We classified individuals into two age groups (immature or adults) by estimating their size relative to a close object when it was possible, or by the size and shape of the head, which is larger and well differentiated from the body in older females. Snakes were designated as aquatic if they were photographed in water or if they had a wet body and were in close vicinity of a water body (*e.g.*, riverside), or terrestrial when they were photographed on land. When none of these criteria applied, we marked the habitat as unknown.

### Environmental variables

We used the *getMoonIllumination* function from the R package *suncalc* (Thieurmel & Elmarhraoui, 2022) to calculate the moonlight fraction, and we also added information regarding the presence of the moon in the sky (if it had risen or not) at the time of the observation. Information regarding the mean temperature of the day (T_24 h_) was obtained from the Open-Meteo.com Weather API (Zippenfenig, 2023) using the default “Best match” option, that uses ERA5 (Hersbach et al., 2020) and ERA5-Land (Muñoz Sabater, 2019) models seamlessly. We used this as a proxy, since data on air, substrate, or body temperature of the individuals at the time of observation were not available.

### Statistical analyses

To check for differences between body patterns regarding T_24 h_, we conducted a one-way ANOVA test, followed by pairwise comparisons using *post-hoc* t-tests with a Holm adjustment for *p*-values. We followed this, with a Kruskal-Wallis H test (values were not normally distributed) on a subset of data situated in the latitudinal range 43–47, where all the phenotypes are encountered in sympatry. The Mann–Whitney U-test was used to examine differences in T_24 h_ between crepuscular and nocturnal observations, and for differences concerning moonlight fraction, given that the values were not normally distributed. Additionally, we explored significant associations between moon presence and age class, as well as moon presence and the two classes of nighttime activity using chi-square tests of independence. All statistical analyses were performed using R Statistical Software v4.2.2 (R Core Team, 2022) with the *ggstatsplot* package (Patil, 2021). The distribution map of nighttime activity, and phenotypes was created using ArcGIS Pro 3.1.3 (Esri, Redlands, CA, USA).

## Results

**Figure 2:**
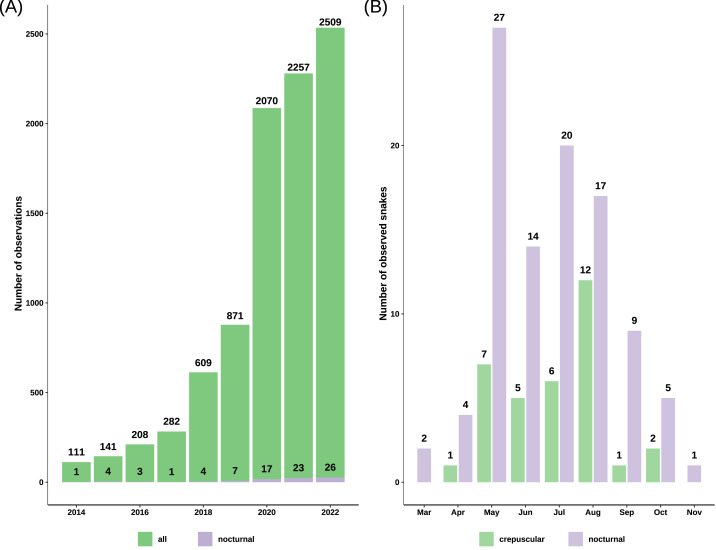
Barplots of grass snake records. (A) Total observations from iNaturalist *versus* the number of nocturnal records from iNaturalist spanning 2014 to 2022, highlighting a notable increase post-2019. (B) Total count of crepuscular and nocturnal snakes per month, revealing a concentration of observations predominantly between May and August.

We obtained 9745 records of grass snakes from iNaturalist, of which 93 were of crepuscular and nocturnal active individuals. After inclusion of our personal observations (22) and the ones obtained from Observation.org (12), the total number of records with nighttime activity increased to 127 observations (Data S2): 33 crepuscular (25.98%) and 94 nocturnal (74.02%). The total number of snakes was 34 crepuscular snakes and 99 nocturnal snakes, as some observations included more than one active individual. Most of the crepuscular and nocturnally active grass snakes from iNaturalist were recorded after 2019 (71.43%) with the platform becoming more popular (Fig. 2). The first observation of nocturnal activity for the grass snake dates back to August 5th 1992, and starting from 2010 there has been at least one observation every year, with the exception of 2013 when no nocturnal record exists.

**Figure 3:**
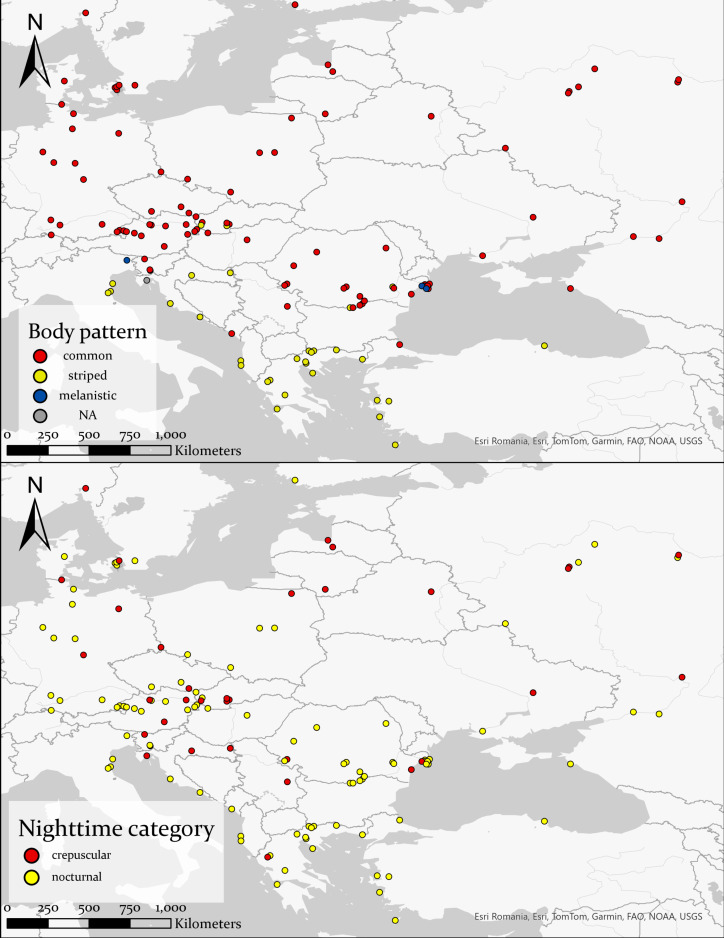
Distribution of different body patterns in crepuscular and nocturnal grass snakes (top half), and distribution of the categories of nighttime activity (bottom half). Basemap Source credit: Esri Romania, Esri, TomTom, Garmin, FAO, NOAA, USGS.

Nocturnal activity was recorded from March 12 to November 5, with most observations occurring between May and August (81.2%) (Fig. 2). Out of 127 observations, 91 snakes had a common morph, 31 had stripes, four were melanistic and for one the body pattern could not be determined because of the poor image quality (Fig. 3). We recorded 88 adults and 35 immatures with crepuscular or nocturnal activity, and we couldn’t determine the age class for ten snakes. Of the snakes observed, 72.93% were found in terrestrial habitats, 21.93% were in aquatic habitats, and the habitat of eight individuals could not be determined from the images.

**Figure 4:**
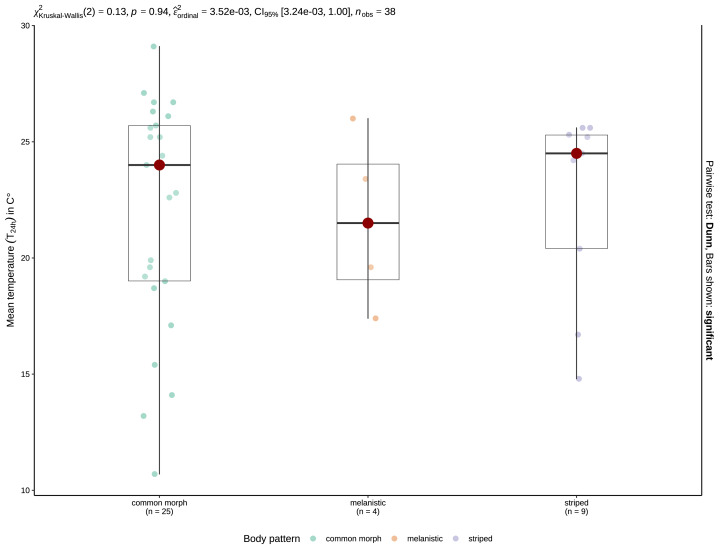
Boxplots of the T_24 h_ for crepuscular and nocturnal observations of common morph (left), melanistic (middle), and striped (right) grass snakes. The red spot indicates the median value. The results of the Kruskal-Wallis test are displayed as the top inset.

Mean T_24 h_ when snakes were active was 20.01 ±0.48 °C (range: 4.7–31 °C, *n* = 127) for crepuscular and nocturnal observations pooled. With the data separated by type of activity, mean T_24 h_ was 19.75 ±0.96 °C (4.7–24.7 °C, *n* = 33) for crepuscular grass snakes, and 20.1 ±0.56 °C (9.2–31 °C, *n* = 94) for nocturnal individuals. A Mann–Whitney U-test, revealed no significant difference in temperature for 24 h between crepuscular (*Mdn* = 20.1) and nocturnal (*Mdn* = 19.9) observations (*U* = 1552.5, *n*_1_ = 33, *n*_2_ = 94, *z* = 0.005, *p* = 0.996). Mean T_24 h_ was 21.6 ±1.92 °C (17.4–26 °C, *N* = 4) for melanistic, 19.18 ±0.58 °C (4.7–30.7 °C, *N* = 91) for common morph, and 22.19 ±0.83 °C (10.9–31 °C, *N* = 31) for nocturnally active striped individuals. A one-way ANOVA revealed that there was a statistically significant difference in mean T_24 h_ between groups, *F* (2,123) = 3.94, *p* = 0.022. Post-hoc tests indicated that striped *N*. *natrix* are active during the nighttime at higher mean T_24 h_ than common morph snakes, *p* = 0.021. A Kruskal-Wallis test (H(2) = 0.13, *p* = 0.94) shows no significant difference regarding T_24 h_ when geographically constrained for the possibility of sympatry (Fig. 4).

A chi-square test of independence showed that there was no significant association between moon presence and Age class *X*^2^ (1, *N* = 112) = 0.198, *p* = 0.656. Also, we did not find any significant association between the type of nocturnal activity (*i.e.,* crepuscular or nocturnal) and moon presence *X*^2^ (1, *N* = 119) = 0.0006, *p* = 0.981. A Mann–Whitney U-test conducted only for observations where the moon was present (*N* = 56) showed no significant difference in the illuminated fraction of the moon between crepuscular (*Mdn* = 0.45) and nocturnal (*Mdn* = 0.7) observations (*U* = 252, *n*_1_ = 15, *n*_2_ = 41, *z* = 1.018, *p* = 0.309).

## Discussion

Several species of primarily diurnal European snakes have been shown to exhibit some form of crepuscular and/or nocturnal activity (Wareham, 1998; Brito, 2003; Michel-Jean, 2013; Zadravec & Koren, 2017; Mattea & Allain, 2020; Dyugmedzhiev et al., 2020; Dyugmedzhiev, 2021; Sahlean et al., 2021) and especially in natricine snakes such as the viperine water snake (Hailey & Davies, 1987; Jaén-Peña & Pérez-Mellado, 1989; Scali, 2011) and the dice snake (Moller, 1990; Scali, Dimitolo & Montonati, 2001; Kreiner, 2007; Mebert et al., 2011; Tuniyev et al., 2011; Scali, 2011). We provide convincing evidence of extensive nocturnal activity in another member of the Natricinae subfamily, the common grass snake (*Natrix natrix*). Although not as frequent as in the other species from the genus *Natrix,* records of nocturnal activity also exist in the grass snake (*N. natrix sensu lato*), best illustrated by the more predominant night movements of Sardinian subspecies *N. helvetica cetti* (Capula, Rugiero & Luiselli, 1994). There is also a mention of minor nocturnal activity in grass snakes from central Germany as radio-tracked individuals were found in water after hot days (Mertens, 1994). Subsequent nighttime checks of individuals that were on land could not provide any evidence of activity (Mertens, 1994). Nocturnal habits are quite common in the dice and viperine snakes from Southern Europe, where climate is generally warmer than in the rest of their range (Jaén-Peña & Pérez-Mellado, 1989; Mebert et al., 2011; Scali, 2011). Most individuals found active during the night were recorded mainly in water, even when temperatures were below 15 °C (Hailey & Davies, 1987; Capula, Rugiero & Luiselli, 1994), while in temperate regions, nocturnality is usually observed during nights when temperatures exceeded 25 °C (Mebert et al., 2011).

The observations in our database cover the entire activity period of the common grass snake and most individuals were recorded between May and August, when the species is most active across its distribution range. A large number of nocturnal individuals were recorded during the month of May, coinciding with a peak in activity as a result of mating (Fuhn & Vancea, 1961; Speybroeck et al., 2016; Sahlean, Strugariu & Gherghel, 2023). Snakes were found active over a wide range of mean daily temperatures (4.7–31 °C), without any significant difference in temperature averages between days in which snakes were active during the crepuscular and nocturnal observations. Grass snakes are known to exhibit a generalist behavior when it comes to temperature-dependent activity and other studies have shown that they can be active at temperatures higher than 6 °C , but they tend to prefer temperatures above 18 °C (Mertens, 1994).

Striped *N. natrix* were found to be active after days with a higher mean temperature compared to the common morph. Indeed, this was expected, as this phenotype is most frequent in the southern part of the species’ distribution range (Fritz & Ihlow, 2022). In the southern Balkans, *N. natrix moreotica*, a subspecies often characterized by two dorsolateral lines (Speybroeck et al., 2016; Asztalos et al., 2021) is present. Moreover, the average annual temperature in the southern Balkans is 4–6 °C higher than in central Europe (World Bank, 2021), where most of the common morph records were observed. After adjusting for the uneven ranges of the studied morphs, further analyses revealed no significant inter-morph differences in T_24 h_. Thus, the assumption that striped individuals might favor higher temperatures during nighttime activities doesn’t hold true in areas where the climate is similar and different phenotypes coexist. We suggest a standardized approach, involving local populations containing all three morphs, with a protocol covering both day and night surveys, for future studies. This approach can determine if coloration affects nighttime activities adaptively and address questions about temperature preferences for each morph in various daily activity patterns. Our results suggest that the moon has no discernible effect on the activity of crepuscular or nocturnal grass snakes. Additionally, we found no significant associations between moon presence and age class. Even when the moon was present at the time of the observation, there was no significant difference in lunar illumination between crepuscular and nocturnal activity. We suggest that further research on this subject be conducted within a systematic framework, as our study relied on observational data collected in a non-standardized manner. Previous research suggests a complex relationship between the moon and snake activity, with varying responses in different snake species. Florida Cottonmouths (*Agkistrodon conanti*) displayed a higher foraging rate during full moon nights, despite the presumed increased risk of predation, suggesting that prey availability and detectability might be more influential than the risk of predation due to increased visibility (Lillywhite & Brischoux, 2012), whereas adult prairie rattlesnakes (*Crotalus viridis viridis*) reduced their activity in higher levels of moonlight, but juveniles did not exhibit the same behavior (Clarke, Chopko & Mackessy, 1996).

We observed a rise in nocturnal records on iNaturalist starting with the year 2020. One possible explanation for this trend is the steady increase in the number of users and observations each year. For instance, in 2017, there were 239 000 active users, which grew to more than 1.8 million in 2020 and exceeded 3.1 million in 2022. The number of crepuscular and nocturnal observations is rather small compared to the vast dataset obtained from iNaturalist; however, it is sufficient to provide an overall understanding of its distribution, as our results cover a significant portion of the known distribution of the common grass snake (Fig. 3). Blais & Shaw (2018) reported similar results using data from iNaturalist, with less than 1% of their observations showing nocturnal activity in a primarily diurnal species, the desert iguana (*Dipsosaurus dorsalis*).

We are just beginning to realize the benefits of citizen science programs in researching populations, communities, and ecosystems, or early detection of potentially invasive species (Bonney et al., 2009; Dickinson, Zuckerberg & Bonter, 2010; Strugariu, 2023). Herpetology, in particular, reaps the rewards from the use of citizen science, especially considering the cryptic behavior exhibited by many species (Tiago, Pereira & Capinha, 2017) and the challenges in assessing their behaviors or population trends (Santos et al., 2022). This is not the first instance of citizen science platforms, such as iNaturalist, being used to gain knowledge about grass snakes. In recent years, georeferenced images from these platforms have proven to be extremely valuable in providing important morphological and distributional data related to grass snakes (Fritz & Ihlow, 2022; Fritz, Grismer & Asztalos, 2023; Jablonski et al., 2023).

## Conclusions

Our study expands our understanding of nocturnal activity in the common grass snake (*N. natrix*), revealing that this is more widespread than previously known, with observations spanning almost its entire distribution range. Although a primarily diurnal species, we suggest extending search efforts through the night, especially in summer months with hot days, as it was suggested by (Capula, Rugiero & Luiselli, 1994). While we found no significant impact of moon presence and moon illumination on nighttime activities, we advocate for additional systematic research to be conducted both in the field (Lillywhite & Brischoux, 2012) and in a controlled environment (Clarke, Chopko & Mackessy, 1996). Moreover, future studies could delve into identifying the primary drivers of nocturnal activity in *N. natrix* and explore its benefits in terms of prey availability and the potential risks involved. This deeper understanding could shed light on the ecological significance of nocturnal activity in diurnal species.

Finally, our research underscores again the immense value of citizen science platforms, such as iNaturalist, as invaluable resources for biological studies. These platforms, with their ever-growing user base of nature enthusiasts, provide a unique opportunity to uncover unusual life traits and behaviors in common species. Although these data are non-systematically recorded and they should be analyzed with caution, in most cases the benefits outweigh the costs. The sheer number of observers covering both space and time cannot be replicated in a scientific protocol due to logistical constraints, making citizen science data an invaluable resource for a wide range of studies.

##  Supplemental Information

10.7717/peerj.17168/supp-1 Data S1 Raw data (all records of Natrix natrix until February 22, 2023) extracted from iNaturalist

10.7717/peerj.17168/supp-2 Data S2 Final dataset comprising of nocturnal and crepuscular observations of Natrix natrix (comprising iNaturalist, Observation.org and authors’ personal observations), together with biological and environmental variables

10.7717/peerj.17168/supp-3 Supplemental Information 3 R script used for statistical analysis and for creating figures

## Additional Information and Declarations

### Data Availability

The following information was supplied regarding data availability: The raw data and R script are available in the Supplementary Files.
